# Variations of nociception level (NOL^®^) measurements during robot-assisted laparoscopic prostatectomy – a monocentric retrospective analysis

**DOI:** 10.1186/s12871-025-03397-0

**Published:** 2025-10-22

**Authors:** Julia Heiden, Jonas Hoefermann, Katharina Hoeter, Jens Kamuf, Robert Kuchen, Miriam Renz, Robert Ruemmler, Alexander Ziebart

**Affiliations:** 1 Department of Anaesthesiology, University Medical Center Mainz, Mainz, Germany; 2 Institute for Medical Statistics, Epidemiology and Informatics, University Medical Center Mainz, Mainz, Germany

**Keywords:** Nociception level index, Nociception monitor, Anaesthesia, Robot-assisted surgery, Non-invasive monitoring

## Abstract

**Background:**

Inadequate analgesia during anaesthesia is associated with a range of complications. While anaesthesiologists routinely monitor the depth of anaesthesia and neuromuscular blockade, no system currently in routine clinical use provides an objective assessment of adequacy of anti-nociception. Although various monitoring systems have been developed in recent years, their impact on the optimization of analgesic therapy remains uncertain. Moreover, the influence of perioperative surgical and non-surgical procedures and events on the measured parameters is not yet fully understood. Nonetheless, this knowledge is essential for the accurate interpretation and effective clinical application of these emerging monitoring technologies.

**Methods:**

Thirty-three patients undergoing robot-assisted laparoscopic prostatectomy using the da Vinci Surgical System were retrospectively analysed. At five specific stimuli (gastric tube placement, urinary catheter placement, initiation of capnoperitoneum, transition to the steep Trendelenburg position and administration of sufentanil) NOL^®^-Index, bispectral index (BIS™), heart rate and mean arterial blood pressure were measured after one, three and five minutes.

**Results:**

We noticed a significant increase in NOL^®^-Index with capnoperitoneum (Beta 14.22, *p* < 0.001), while the NOL^®^-Index decreased after steep Trendelenburg position (Beta − 8.89, *p* = 0.002) and sufentanil application (Beta − 17.67, *p* < 0.001). No significant changes were observed during gastric tube placement and urinary catheter insertion. The BIS^™^ analysis showed no relevant deviation during anaesthesia.

**Conclusion:**

The NOL^®^-Index showed characteristic changes during robot-assisted laparoscopic prostatectomy. Our study shows plausible results that can be used as a basis for future prospective studies to evaluate the clinical relevance of nociceptive monitoring.

## Introduction

While anaesthesiologists routinely use methods such as bispectral index (BIS™) monitoring to evaluate depth of anaesthesia and prevent awareness or neuromuscular monitoring to control muscle relaxation as a standard of care, there are still no devices available to objectively assess nociception in patients under general anaesthesia. [1, 2] Nociception, in this context, refers to the neurophysiological process by which potentially tissue-damaging stimuli (mechanical, thermal, or chemical) are detected by specialized sensory receptors (nociceptors) and transmitted via peripheral and central neural pathways. Importantly, nociception must be distinguished from pain: whereas nociception encompasses only the encoding and transmission of noxious input, pain constitutes the conscious, subjective perception that arises from higher-order central processing. To adequately address this process and mitigate its adverse physiological and clinical consequences, appropriate analgesic management is required. [3]

Analgesic management relies on indirect parameters such as blood pressure, heart rate (HR) and breathing patterns, which can be influenced by various intraoperative factors, including capnoperitoneum, patient positioning, volume shifts (e.g. blood loss), and medications such as beta-blockers or catecholamines. [4–6] Inadequate analgesia is associated with various complications. While hyperanalgesia can cause haemodynamic instability, prolong extubation time and increase the risk of delirium. Insufficient analgesia may heighten the stress response and postoperative pain, leading to a higher incidence of postoperative complications like nausea and vomiting. [6–8]

In recent years, several devices have been developed to fill this gap, such as the PMD-200-Nociception Monitor (NOL^®^; Medasense, Ramat Gan, Israel). This non-invasive device uses a finger clip that records photoplethysmography, galvanic skin response, peripheral temperature, and accelerometry. From these signals, an integrated algorithm derives several physiological variables associated with nociception, including pulse rate, pulse rate variability, pulse wave amplitude, skin conductance, peripheral temperature, and movement. These parameters are combined into the NOL^®^-Index, a dimensionless score ranging from 0 to 100. Values between 10 and 25 are considered indicative of adequate analgesia. [9] There are multiple studies demonstrating the effectiveness of the NOL^®^-Index; however, there is currently no conclusive evidence to support the hypothesis that its use reduces opioid requirements and postoperative complications. Some studies have reported a reduction in opioid administration per hour with NOL^®^-Monitoring, though not in the total amount, while others suggest a 20% decrease in remifentanil consumption. [10–12] A meta-analysis showed that NOL^®^-Monitoring was associated with a shorter extubation time. [8] However, the clinical relevance of these findings remains uncertain.

Designing and conducting future prospective trials requires a fundamental understanding of the nociception profile throughout surgical procedures and identifying perioperative events that influence it, such as patient positioning, capnoperitoneum or catecholamine administration. A 30% intraoperative reduction in remifentanil administration during laparoscopic colorectal surgery under deep neuromuscular blockade has been observed when analgesia was guided by the NOL^®^ -Index. [13] Additionally, an intravenous bolus of phenylephrine at 1 µg·kg⁻¹ was associated with a mean change in the NOL^®^-Index of 2.9, which, while statistically significant, appears to lack clinical relevance. [13, 14] This understanding is essential for accurately interpreting results and can directly impact clinical decision-making.

This study focused on robot-assisted laparoscopic prostatectomy (RALP) due to the relatively standardised perioperative protocols and a rapid increase in cases over the past years. [5, 15] Hence, this study aimed to evaluate the impact of characteristic perioperative events on the NOL^®^-Index and other standard monitoring parameters during those minimally invasive interventions.

## Materials and methods

### Ethics

This analysis was registered under the identifier DRKS00029120, with ethics committee approval (No. 2021–16201) obtained from the regional ethics committee of Rhineland Palatinate, Mainz, chaired by Dr. Stephan Letzel. Individual consent requirements were waived by the committee.

### Patients

The data of 33 male patients undergoing robot-assisted, minimally invasive prostatectomy using the da Vinci Xi Surgical System (Intuitive Surgical Inc., Sunnyvale, CA, USA) between the 1 st of May and the 31 st of August 2023 at the University medical center of the Johannes-Gutenberg University in Mainz, Germany were retrospectively analysed. All patients received general anaesthesia. No fixed treatment protocol was specified, leaving all therapeutic decisions at the discretion of the trained professional conducting the anaesthesia. We included all patients, who were monitored using the NOL^®^-Index according to the hospitals internal standard within the aforementioned timeframe and excluded patients with conditions that could interfere with the NOL^®^-System. Such conditions were arrhythmia, cardiac pacemaker implantation prior to the intervention or severe hypothermia. All patients were classified as ASA status 2 and 3. The median age was 65 ±6 years.

### Monitoring

All patients received standardised haemodynamic monitoring (non-invasive blood pressure measurements, HR, oxygen saturation). Nociception and stress levels were detected using the PMD-200 Nociception Level (NOL^®^) measurement device (PMD-200; Medasense Biometrics Ltd., Ramat Gan, Israel) while the depth of anaesthesia was monitored via the bispectral-index monitoring system (BIS^™^-monitoring system, Medtronic, Minneapolis, USA).

At the time of five distinct perioperative procedures, we measured the NOL^®^-Index, the BIS^™^-Index, the HR and the mean arterial blood pressure (MAP) at baseline (0), and one, three and five minutes after the event respectively. These procedures were gastric tube placement, urinary catheter placement, initiation of capnoperitoneum, transition to the steep Trendelenburg position and administration of sufentanil. Each bolus application of sufentanil was evaluated separately. Boluses containing between 5 and 15 micrograms of sufentanil were administered.

### Statistics

The statistical analyses were performed using GraphPad Prism Version 10.3.1 (GraphPad Software LLC, Boston, MA, USA). A retrospective secondary analysis was conducted on prospectively approved, anonymized quality assurance data. Due to the pilot nature of this analysis and the absence of prior statistical planning, the evaluations are primarily descriptive. All data were anonymised to minimise selection and reporting bias. Mixed linear regression models were used to analyse the differences in measurements. This approach was chosen in order to account for the repeated-measures design and the hierarchical structure of the data, which comprised multiple observations within individual patients. These models enabled both fixed effects (e.g. type of event) and random effects (e.g. inter-individual variability) to be included, thereby improving the robustness and interpretability of the statistical results. The Beta Coefficient (B) is defined as the slope of the regression line, representing the effect of an independent variable on the dependent variable. A positive B-value indicates a positive relationship while a negative B-value indicates a negative. The larger the B-value is, the stronger the effect is. The confidence interval (CI) was set at 95%. This indicates that, with a probability of 95%, the confidence interval includes the true value of B. A confidence interval that does not contain the value 0 is indicative of a significant correlation. P-values < 0.05 were considered statistically significant [16].

## Results

**Fig. 1:**
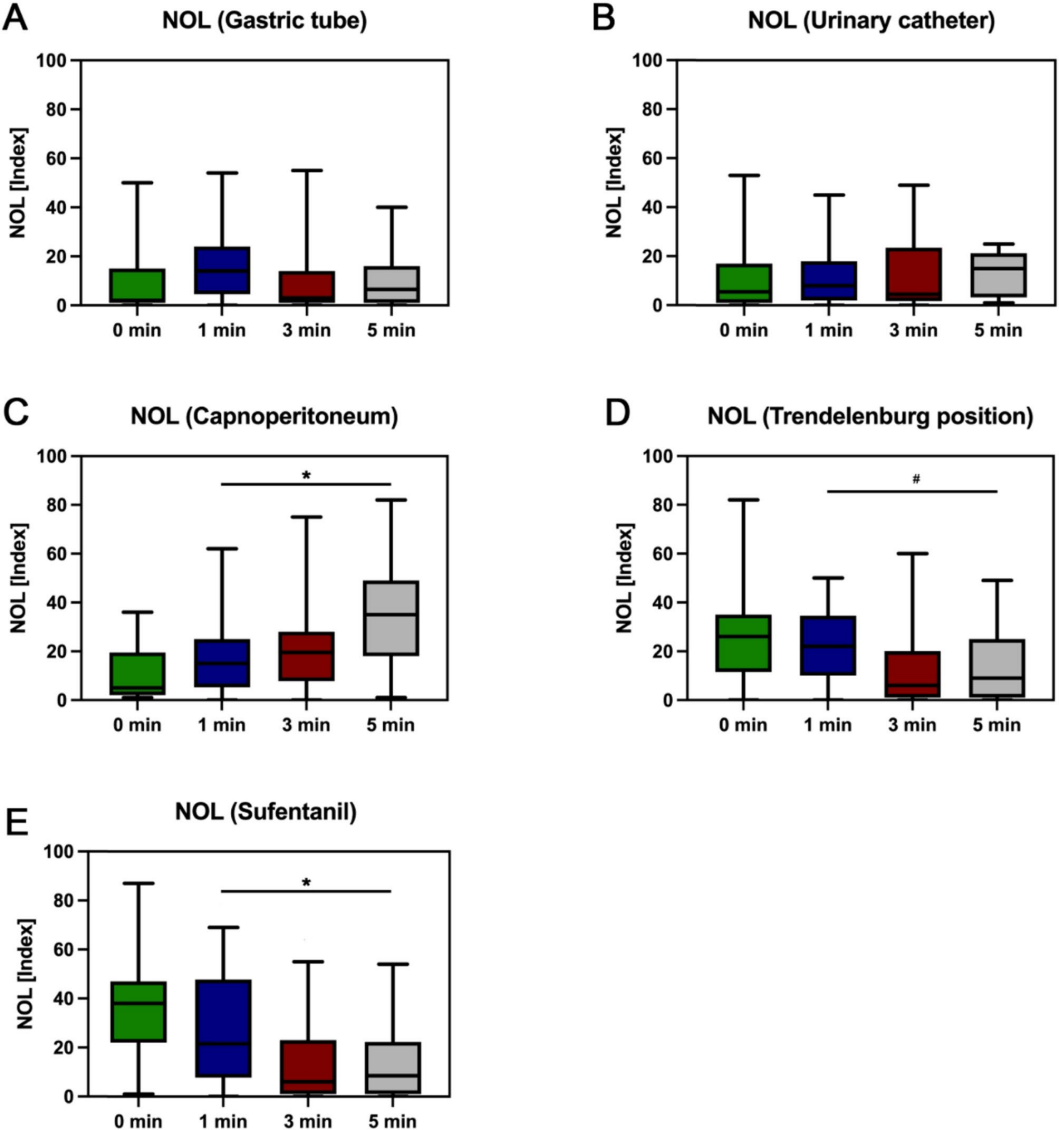
Variations of the NOL^®^-Index at gastric tube placement (**A**, *n* = 33), urinary catheter placement (**B**, *n* = 32), initiation of capnoperitoneum (**C**, *n* = 32), transition to the steep Trendelenburg position (**D**, *n* = 33) and administration of sufentanil (**E**, *n* = 42) after one, three and five minutes compared to baseline (^#^ = *p* < 0.05, *= *p* < 0.001). Mixed linear regression models were used to analyse differences in measurements at the given time points compared to the baseline

After gastric tube placement, we observed no significant changes in NOL^®^-Index (Beta 2.65, 95% CI −1.76–7.06, *p* = 0.2) (Fig. 1), BIS^™^ (Beta 0.55, 95% CI −1.30–2.39, *p* = 0.6) and MAP (Beta − 4.39, 95% CI −9.66–0.87, *p* = 0.1). The only significant change was noted in HR (Beta − 2.21, 95% CI −4.03 - −0.40, *p* = 0.017). With placement of urinary catheter there were no significant changes at all: NOL^®^-Index (Beta − 0.03, 95% CI −4.18–4.12, *p* > 0.9) (Fig. 1), BIS™ (Beta − 0.41, 95% CI −3.87–3.05, *p* = 0.8), HR (Beta 0.35, 95% CI −1.06–1.75, *p* = 0.6), MAP (Beta − 0.43, 95% CI −1.78–0.92, *p* = 0.5).

We noticed significant changes in NOL^®^-Index (Beta 14.22, 95% CI 8.21–20.23, *p* < 0.001), HR (Beta 7.26, 95% CI 4.63–9.89, *p* < 0.001) and MAP (Beta 9.0, 95% CI 4.61–13.39, *p* < 0.001) after initiation of capnoperitoneum (Fig. 1) while there was no significant difference in BIS^™^ (Beta 0.57, 95% CI −0.93–2.06, *p* = 0.5).

After transferring the patients into a steep Trendelenburg position there was no significant change in MAP (Beta 2.67, 95% CI −1.88–7.22, *p* = 0.2) but in all the other parameters: NOL^®^-Index (Beta − 8.89, 95% CI −14.52 - −3.26, *p* = 0.002) (Fig. 1), BIS^™^ (Beta 4.19, 95% CI 1.59–6.8, *p* = 0.002) and HR (Beta − 2.76, 95% CI −5.37 - −0.5, *p* = 0.039).

The application of sufentanil led to significant changes in all observed parameters: NOL^®^-Index (Beta − 17.67, 95% CI −22.57 - −12.77, *p* < 0.001) (Fig. 1), BIS^™^ (Beta − 3.27, 95% CI −4.82 - −1.72, *p* < 0.001), HR (Beta − 2.75, 95% CI −4.48 - −1.03, *p* = 0.002) and MAP (Beta − 4.82, 95% CI −7.35 - −2.29, *p* < 0.001).

Individual parameter values are presented in Table 1.

**Table 1:** Evaluation of NOL^®^-Index, BIS^™^, HR and MAP parameters during characteristic perioperative events over five minutes

	Parameter	0 min	1 min	3 min	5 min	
Gastric tube placement	NOL^®^-Index	9 ± 13	16 ± 14	10 ± 13	10 ± 11	
	BIS™	43 ± 12	44 ± 13	44 ± 12	42 ± 12	
	HR (bpm)	56 ± 11	56 ± 11	53 ± 11	52 ± 10	#
	MAP (mmHg)	91 ± 22	87 ± 22	87 ± 21	84 ± 16	
Urinary catheter placement	NOL^®^−Index	12 ± 13	10 ± 10	12 ± 14	13 ± 8	
	BIS™	42 ± 16	42 ± 13	36 ± 7	36 ± 9	
	HR (bpm)	51 ± 9	51 ± 9	53 ± 13	49 ± 8	
	MAP (mmHg)	80 ± 11	79 ± 11	78 ± 11	79 ± 8	
Capnoperitoneum	NOL^®^−Index	10 ± 10	18 ± 16	21 ± 17	35 ± 21	*
	BIS™	42 ± 12	42 ± 12	42 ± 12	43 ± 13	
	HR (bpm)	51 ± 9	53 ± 10	57 ± 11	63 ± 12	*
	MAP (mmHg)	79 ± 11	82 ± 12	88 ± 16	92 ± 20	*
Steep Trendelenburg position	NOL^®^−Index	26 ± 18	23 ± 14	12 ± 15	15 ± 15	#
	BIS™	39 ± 9	42 ± 13	44 ± 13	43 ± 13	#
	HR (bpm)	63 ± 12	60 ± 13	61 ± 12	61 ± 11	#
	MAP (mmHg)	99 ± 19	100 ± 22	104 ± 14	101 ± 11	
Sufentanil application	NOL^®^−Index	35 ± 19	25 ± 20	14 ± 17	13 ± 15	*
	BIS™	48 ± 13	46 ± 11	44 ± 12	44 ± 12	*
	HR (bpm)	69 ± 12	68 ± 9	65 ± 11	65 ± 10	#
	MAP (mmHg)	104 ± 16	104 ± 18	100 ± 17	94 ± 13	*

All values are presented in Mean ± Standard deviation

*NOL*^®^-*Index* Nozizeption Level Index, *BIS*™ Bispectral index, *HR* Heart Rate, *MAP* Mean arterial pressure

# = *p* < 0.05, * = *p* < 0.001 compared to baseline

## Discussion

The objective of this study was to evaluate the influence of characteristic perioperative events on the NOL^®^-Index and other standard monitoring parameters during RALP. We noticed significant changes in the NOL^®^-Index with initiation of a capnoperitoneum, Trendelenburg positioning and the application of sufentanil.

Following the placement of a gastric tube, the only significant change that was identified was a decrease in HR. The gastric tube was placed manually - the use of a laryngoscope and Magill forceps was not required. The phenomenon of HR decrease during placement can be adequately explained by the laryngocardiac reflex, as laryngeal stimulation leads to bradycardia due to a vagal response. [17] We did not observe significant changes in NOL^®^-Index, BIS™ and MAP after placement of gastric tube and no changes at all after the placement of the urinary catheter. We suspect this could be due to the recent induction of anaesthesia.

The initiation of capnoperitoneum is expected to act as a highly nociceptive stimulus. [18, 19] In our study we noticed significant changes in the NOL^®^-Index, HR, and MAP, without significant change in BIS^™^ values. This suggests that the depth of anaesthesia remains constant and the increase in NOL^®^-Index cannot be explained by insufficient depth of anaesthesia. Capnoperitoneum is known to cause characteristic changes in the haemodynamic reactions, especially during the initial phase, when the venous return increases due to compression of splanchnic vessels. Concurrently, the rise in intra-abdominal pressure activates the sympathetic nervous system, while the rise in arterial pCO_2_ affects pulmonary and peripheral vascular tension. [5] These physiological changes in the haemodynamic regulation might have an influence on the NOL^®^-Index, as Raft et al. were able to show an increase in NOL^®^-Index measurements after the administration of phenylephrine. [13] While this result was statistically significant, it is likely to be of minimal clinical relevance due to the observed small effect size. It is important to consider that the PMD-200-Nociception Monitor also analyses movement, temperature and skin conductivity. At this point, our results are in line with those of Ruemmler et al., who also showed a significant increase in NOL^®^ during skin incision. [20] Therefore, we estimate that the NOL^®^-Index is effectively able to reliably indicate nociception at this point. The NOL^®^-Index demonstrated a decline after the adoption of a steep Trendelenburg position. The mean NOL^®^-Index at the baseline was 26. The range of 10 to 25 is considered to be adequate analgesia. [9] It is possible that simultaneous positioning and opioid application influenced our results, as we assume that Trendelenburg positioning does not cause nociceptive stimuli. The mean BIS^™^ increased from 39 ± 9 to a maximum of 44 ± 13, which reaches statistical significance, but clinical relevance is questionable. We would have suspected an increase in MAP when transferring patients to the steep Trendelenburg position, but our findings revealed no significant alterations. The reason for this may be that the venous return is not further improved by the Trendelenburg position during the capnoperitoneum. HR decreased significantly as it is known for Trendelenburg positioning. [5, 21]

All measured parameters decreased significantly after application of sufentanil. Sufentanil is a highly potent opioid which is used in balanced general anaesthesia. It is to be expected that its application leads to decreases in the NOL^®^-Index due to its analgesic effects. [5, 21] These findings highlight the potential of different factors that affect the capacity of the NOL^®^-Index to detect subtle nociceptive responses that might otherwise go unnoticed. Insights into the interaction between surgical conditions (such as capnoperitoneum) and NOL^®^-Index. Such protocols could support more precise titration of opioid administration and ultimately improve perioperative pain management and patient outcomes.

This study has some limitations. Its retrospective design and the relatively small sample size limit the generalizability of the findings and increase the risk of both Type I and Type II errors, especially in the context of repeated measurements and multiple comparisons. No formal adjustments were made for potential confounders such as anaesthetic depth (BIS™), baseline hemodynamics, or opioid administration patterns, which were documented but not included in the statistical models. As no power analysis was performed, the results should be interpreted as exploratory and hypothesis-generating.

Anaesthetic management was not standardized beyond institutional guidelines, reflecting routine clinical practice. While this may introduce heterogeneity and potential confounding factors, it could also enhance the real-world applicability of the findings. NOL^®^-Index values were recorded within predefined time windows, allowing partial comparability despite variability in anaesthesia techniques. However, residual confounding cannot be excluded.

The exclusive focus on RALP improves data homogeneity but limits transferability to other surgical procedures. The five-minute measurement windows post-intervention restrict insights into longer-term outcomes. Furthermore, the uniform timing across physiologically diverse events is a methodological limitation. In future prospective studies we would like to incorporate continuous monitoring, broader physiological metrics, and adaptive analysis models.

Finally, the lack of postoperative outcome data (e.g. pain scores, opioid use) limits the clinical interpretability of intraoperative NOL^®^-Index changes. Prospective trials are needed to assess its predictive and clinical value in the perioperative setting.

## Conclusions

In conclusion, we observed characteristic changes in the NOL^®^-Index during robot-assisted laparoscopic prostatectomy. Specifically, the NOL^®^-Index increased significantly with the initiation of capnoperitoneum, decreased after Trendelenburg positioning, and showed a marked reduction following sufentanil administration. These findings suggest that the NOL^®^-Index is responsive to distinct perioperative nociceptive stimuli and may detect subtle analgesic effects even when standard monitoring parameters remain stable. Our study provides plausible results that could serve as a basis for future prospective studies to further establish the clinical relevance of nociceptive monitoring.

## Data Availability

The original contributions presented in this study are included in the article. Further inquiries (raw data) can be directed to the corresponding author.

## Abbreviations

B: Beta Coefficient
BIS™: Bispectral index
Bpm: Beats per minute
HR: Heart rate
MAP: Mean arterial blood pressure
mmHg: Millimetres of mercury
NOL^®^: Nociception Level
CO: Cardiac output
pCO: Partial pressure of carbon dioxide
RALP: Robot-assisted laparoscopic prostatectomy
